# Thermal conductance of Teflon and Polyethylene: Insight from an atomistic, single-molecule level

**DOI:** 10.1038/srep41898

**Published:** 2017-02-02

**Authors:** Marius Buerkle, Yoshihiro Asai

**Affiliations:** 1 National Institute of Advanced Industrial Science and Technology (AIST), Research Center for Computational Design of Advanced Functional Materials (CD-FMat), Central 2, Umezono 1-1-1, Tsukuba, Ibaraki 305-8568, Japan

## Abstract

The thermal transport properties of teflon (polytetrafluoroethylene) and its polyethylene counterparts are, while highly desirable and widely used, only superficially understood. Here, we aim therefore to provide rigorous insight from an atomistic point of view in context of single-molecule devices. We show that for vinyl polymers adsorbed on metal-surfaces the thermal transport strongly depends on the properties of the metal-molecule interface and that the reduced thermal conductance observed for teflon derivatives originates in a reduced phonon injection life time. In asymmetric molecules phonon blocking on the intra molecular interface leads to a further reduction of thermal conductance. For hetrojunctions with different electrode materials we find that thermal conductance is suppressed due to a reduced overlap of the available phonon modes in the different electrodes. A detailed atomistic picture is thereby provided by studying the transport through perfluorooctane and octane on a single-molecule level using first principles transport calculations and nonequilibrium molecular dynamic simulations.

Polytetrafluoroethylene (PTFE) accidentally discovered by Roy Plunkett in 1938 is probably best known under its brand name “Teflon”. While it played a central role in Manhattan Project being the only material which could contain the highly reactive uranium hexafluoride and its use as thermal shielding and insulation in the NASA space program, the most prominent application of “Teflon” which comes to mind is certainly the low friction, hydrophobic, and heat resistance coating of nonstick pans. However, owing to its remarkable properties, PTFE derivatives are nowadays used in a much wider range of applications. Teflon dielectrics are often the material of choice for high performance insulators and shieldings in wires and circuit boards. Especially when also a high chemical and thermal stability is required PTFE is usually superior to polyethylene (PET) based vinyl polymers^1^,^2^,^3^. For example the high thermal stability and its low thermal conductivity make PTFE an excellent high temperature insulation. However, often competing properties are required, while the low friction coefficient is desirable for mechanical applications its high degree of wear and its low thermal conductivity can compromise the applicability. While the mechanical properties, such as the low friction coefficient and high wear^4^, and the dielectric properties^5^ of PTFE are well studied, its low thermal conductivity remains rarely addressed. In general it is difficult to study the thermal transport in bulk materials at an atomistic level. In this work we therefore take a different route, focusing on the thermal conductance of PTFE and PET derivatives in the context of single molecule devices. Especially for low density vinyl polymers we can expect to observe similar transport characteristics as in the single molecule case. Here, by studying the phonon transport through perfluorooctane and octane-based molecular junctions we can elucidate the thermal transport properties of PTFE and PET from an atomistic point of view. This allows us to focus on the role of the metal/molecule interface which is expected to have a decisive role on thermal transport as it has been recently shown that the intrinsic thermal conductance of PTE chains remains, due to its quasi ballistic behavior, very high even for chains with a length of several hundred nanometers^6^,^7^. The considered octane and perfluorooctane derivatives, as displayed in Fig. 1a, are used as prototypical system for PTFE and PTE adsorbed on aluminum surfaces. We find that the thermal conductance of the fluorinated molecule is by around a factor of 2 smaller as compared to octanediol, the reduction is attributed to a reduced injection life time of the phonon modes due to a reduced coupling to the electrodes. Partial fluorination leads additionally to phonon blocking on the molecular bridge reducing the thermal conductance further. We also investigate the influence of the electrode material, replacing one aluminum electrode with lead narrows the energy window available for phonon propagation, which in turn limits the phonon thermal conductance largely by filtering out the molecular vibrations at higher energies. Essentially the thermal conductance will be mainly determined by the interface resistance and the contribution of the intrinsic thermal resistance of the molecular bridge remains comparably small for the molecules studied here and in the limit of infinitely long chains both contribution are expected to be comparable. The thermal transport properties are thereby obtained by atomistic first principles calculations in combination with nonequilibrium Green’s functions and classical nonequilibrium molecular dynamics (NEMD) simulations using accurate reactive force fields.

## Results

### Interface properties and thermal conductance

**Figure 1:**
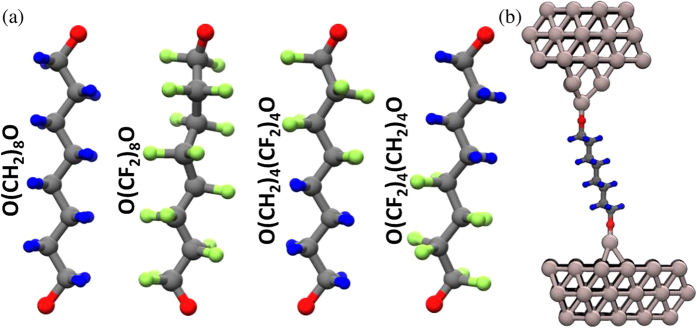
(**a**) Considered octanediol derivatives. (**b**) Example of the STM-like junction geometry.

**Figure 2:**
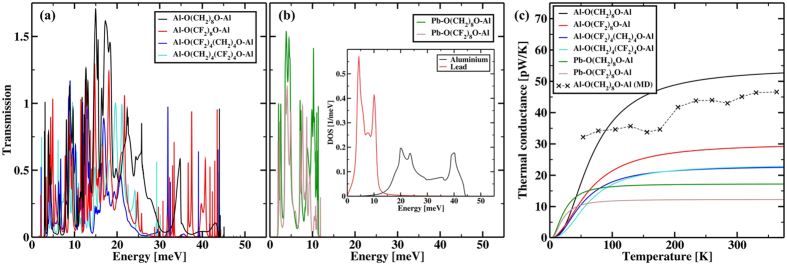
(**a**) Phonon transmission spectra for octane-1,8-diol, hexadecafluoro-1,8-octanediol, as well as for the partially fluorinated molecules connected to aluminum electrodes. (**b**) Phonon transmission spectra using lead instead of aluminum as substrate electrode. Bulk DOS of Aluminum and Lead. (**c**) Thermal conductance of all studied junctions. Solid lines are first principles results, dashed line indicate results obtained from classical RNEMD.

**Figure 3:**
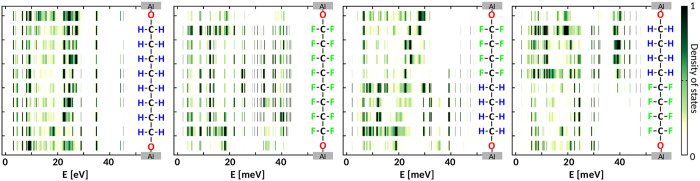
Projected density of states along the CH_2_ (CF_2_) units, where the DOS is normalized to the maximum element of all 4 junctions.

The ballistic thermal conductance of the 4 studied octane-derivatives, 1,8-octanediol O(CH_2_)_8_O, hexadecafluoro-1,8-octanediol O(CF)_8_O, and partially fluorinated O(CH_2_)_4_(CF_2_)_4_O (Fig. 1) is calculated by means of


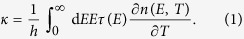


and determined from the energy *E* dependent transmission probability *τ(E*) of the phonons through the junctions, here *h* denotes the Planck constant and *n(E*) = 1/[exp(*E*/(*k*_*B*_*T*)) − 1] is the Bose–Einstein distribution calculated at an average junction temperature *T* where *k*_*B*_ is the Boltzmann constant. The calculated phonon transmission spectra using Al electrodes and the corresponding thermal conductance *κ(T*) is summarized in Fig. 2. While the absolute value of the conductance differs notably for higher temperatures, the overall temperature dependence is similar for all 4 molecules, especially the onset of the saturation of *κ(T*) at around 150 K is similar for all four studied molecules. In the low temperature region up, to 30 K, the thermal conductance of O(CH_2_)_8_O and O(CF_2_)_8_O junctions is comparable. With increasing temperature, however, we observe for O(CH_2_)_8_O a much steeper increase of *κ(T*) with *T*. As we approach the classical limit, the thermal conductance of O(CH_2_)_8_O is around a factor of 2 larger than that of O(CF_2_)_8_O. The thermal conductance of the partially fluorinated molecules is overall further reduced as compared to the fully fluorinated one. Where we consider both possible orientations, i.e., either the fluorinated or the unfluorinated part connected by the STM-like electrode. For both cases the thermal conductance is essentially identical in the classical limit and does also only weakly deviate at lower temperatures. Substituting the light H atoms with the much heavier F introduces additional modes into the energy window defined by the density of states (DOS) of the electrodes, thus one could, contrary to our findings, expect the thermal conductance to increase. To elucidate this counter intuitive behavior we consider the projected DOS along the carbon chain where we sum over the contribution of the three individual atoms of each CH_2_ (CF_2_) unit (Fig. 3). For O(CH_2_)_8_O the vibrational modes below 30 meV are well delocalized in energy space giving rise to the broad peaks observed in the transmission spectra. For higher energies, on the other hand, only a few localized modes are present. For O(CF_2_)_8_O the fluorination introduces a large number of new modes in the transport relevant energy range, they are however only very weakly broadened due to their small injection life time, suggesting a largely reduced coupling to the electrodes. Especially for the modes above 30 meV the reduced injection life time confines the vibrations on the CF_2_ units giving rise to the very sharp resonances in the high energy end of the transmission which only give a negligible contribution to the overall thermal transport. Although that the effect is somewhat reduced for lower energies the resonances in the transmission spectra of O(CF_2_)_8_O are much sharper as compared to O(CH_2_)_8_O resulting in the observed reduction of the thermal conductance for the fluorinated molecule. Partially fluoridating the molecule creates an additionally interface on the molecular bridge and a phonon mismatch between the molecular vibration on the fluorinated and unfluorinated parts of the molecule, as clearly visible in Fig. 3. The phonon blocking at this interface leads to the further reduction of the thermal conductance and is for the most part independent on whether the fluorinated or unfluorinated part is connected to STM-like electrode. Next, the influence of the electrode material is investigated by replacing the substrate material from aluminum to the much heavier lead which decrease the energy of the phonon spectrum notably (Fig. 2b inset). The overlap of the DOS of both electrodes is limited to the low energy end of the Al DOS. Accordingly we observe a finite phonon transmission only within 0 meV to ~15 meV (Fig. 2b). Comparing the thermal conductance of the aluminum junction and the aluminum/lead hetrojunction (Fig. 2c) shows that for low temperatures the thermal conductance of the Pb-octane-Al junctions is somewhat larger due to the steep increase of *κ(T*) with *T*. However, as *κ(T*) saturates for Pb/octane/Al already at 50 *K* the thermal conductance of the Al/octane/Al junction becomes much larger in the classical limit, differing by around a factor of 5 for temperatures above 200 K. Similar to the preceding discussion substituting H with F leads to a further reduction of the thermal conductance, however due to the smaller number of modes the effects is not as pronounced as for the junction with two aluminum electrodes.

### Nonequilibrium temperature profile

To further elucidate the transport properties we calculated the temperature profile over the junction using reverse nonequilibrium molecular dynamic simulations (RNEMD). The temperature profile for a given temperature gradient Δ*T* is connected to the energy transfer per time d*Q*/d*t* and hence to the steady-state thermal conduction *κ* by means of Fourier’s law


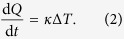


**Figure 4:**
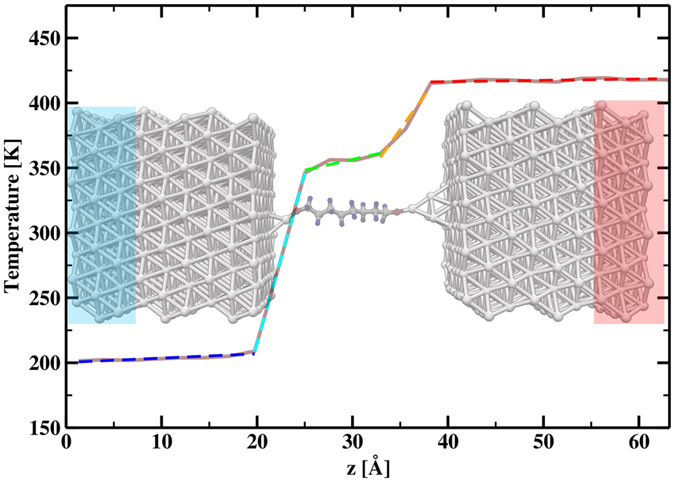
Time-averaged temperature profile over the junction, with linear fits in each distinct spatial region. For simplicity the periodic part of the junction used in the RNEMD approach is omitted.

From the steady-state temperature profile across the junction it is possible to obtain not only the total thermal conductance but also information about the individual spatial regions inside the junction. For an average junction temperature of *T* = 311 K the temperature profile is given in Fig. 4. The largest temperature drop occurs at the two electrode/molecule interfaces, thus the major contribution to *κ* arises from the interfacial conductance and only a small part from the intrinsic thermal conductance of the molecule. For the system studied here the total junction thermal conductance *κ*_tot_ = 1/*R* is determined by the sum *R* = *R*_*L*_ + *R*_*LM*_ + *R*_*M*_ + *R*_*RM*_ + *R*_*R*_ of the individual thermal resistances occurring through the junction, where *R*_*M*_ is the resistance of the molecular bridge, *R*_*L*_ and *R*_*R*_ are the thermal resistances of the left (L) and right (R) electrode, and *R*_*LM*_ and *R*_*RM*_ the corresponding contact resistances at the two electrode/molecule interfaces. In principal an additional contribution arises in the RNEMD approach due to the artificial interface created in the electrodes, this contribution is, however, negligible small. From the temperature profile we can calculate the temperature gradient in each region which allows us to split the total thermal conductance into its individual contributions as summarized in Table 1. As suggested the conductance is mainly determined by the metal/molecule interface, while the intrinsic thermal conductance of the molecule plays only a minor role. The contribution from the electrodes are essentially negligible. The total thermal conductance obtained from adding up the individual contributions is basically identical to the value *κ* = 45.1 pW/K obtained by calculating the thermal conductance directly by means of equation 2. It is also worth noting that the thermal conductance obtained here for aluminum 27480 pW/K agrees very well with recent DFT calculations were a value of *κ*_Al_ = 25200 pW/K was reported^8^. The thermal conductance obtained from RNEMD is compared with the first principles results in Fig. 2c. While for low temperature the RNEMD approach is expected to fail due to missing quantum mechanical effects^9^ we find that for higher temperatures (*T* > 200 K), where the thermal conductance tends to saturate towards the classical limit, the RNEMD results agree very well with the first principles calculations reproducing the absolute values as well as the weak temperature dependence of *κ(T*). Moreover, while in contrast to the purely ballistic first principles results, the RNEMD approach includes anharmonic interaction we do not observe corresponding signatures in *κ(T*), which is consistent with the previously reported weak anharmonicity in alkane chains^10^,^11^ and bulk aluminum^12^ in the studied temperature range.

## Discussion

To summarize, we elucidated the thermal transport through teflon and polyethylene adsorbed on metal surfaces from an atomistic point of view. We find that the thermal conductance of teflon is, consistent with experimental observations, notably suppressed compared to polyethylene. The phonon thermal conductance is thereby essentially determined by the vibrational modes of the molecular conductor, the available phonon density of states of the two electrodes, and the injection life time of the molecular vibrations due to interaction with the electrodes. Here, we observe two competing effects, on the one hand teflon has a much larger number of phonon modes inside the transport relevant energy range, however their injection life time is largely reduced compared to polyethylene due to a weaker coupling to the electrodes. Eventually the latter effects dominates resulting in the reduced thermal conductance of teflon. In the case of asymmetric molecules, which are only partially fluorinated, phonon blocking at the intra molecular interface will lead to a further suppression of the thermal conductance. Additionally to the influence of the interfaces the intrinsic properties of the electrodes are playing an important role, replacing the material of one electrode from aluminum to lead will decrease the thermal conductance considerably be reducing the overlap of the phonon density of states of the two electrodes. To conclude, we want to emphasize that while we have considered here rather short molecules in the context of single molecule devices allowing us to focus on the atomistic details, we can expect, due to the intrinsic quasi ballistic transport through the molecular conductor, that the interface properties dominate even for chains of several 100 nm of length^6^.

## Method

### Vibrational structure

The electronic structure of the molecular junctions is described within density functional theory (DFT) using the PBE level of theory^13^,^14^ and a double zeta basis set^15^,^16^. The second derivative of the total energy with respect to the nuclear displacements and hence the vibrational modes are obtained analytically within density functional perturbation theory (DFPT)^17^,^18^. The total energy is converged to a precision of better than 10^−9^ a.u., while geometry optimizations are performed until the change of the maximum norm of the Cartesian gradient is below 10^−5^ a.u. These tight convergence criteria are necessary to ensure an accurate description of the vibrational properties. All DFT and DFPT calculations were carried out using the quantum chemistry package TURBOMOLE^19^.

### Contact geometries

The contacts are constructed by first relaxing the molecule along with the single Al ad-atom on top of one Al electrode modeling the substrate, then the second electrode modeling an atomic sharp STM tip is attached such that the Al-O distance is similar to the distance obtained in the first step. Next, the molecule along with the Al ad-atom of the substrate and the innermost 4 Al of the tip are again fully relaxed. All other electrode atoms are kept fixed at ideal fcc (111) lattice positions. For the fully relaxed structures we find that the C-C bond length is similar for O(CH_2_)_8_O (1.55 Å) and O(CF_2_)_8_O (1.58 Å), however while for O(CH_2_)_8_O the C atoms all lie within the same plane they are rotated out of the plane for O(CF_2_)_8_O.

### Phonon transport from first principles

The phonon heat current can be obtained in the Landauer-Büttiker picture by^20^,^21^


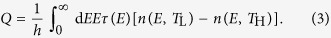


where *τ(E*) is the phonon transmission function and *n(E, T*) = {exp(*E*/*k*_*B*_*T*) − 1}^−1^ is the Bose function, characterizing the phonon reservoirs of the two electrodes at temperatures *T*_*L*_ and *T*_*H*_. In linear response the corresponding thermal conductance is given by


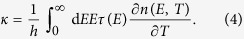


Essentially, the transport is determined by the phonon transmission function *τ(E*) which is here calculated using a Green’s function formalism^21^,^22^,^23^,^24^. Details and a comprehensive discussion of our transport formalism can be found in ref. 24.

### Nonequilibrium molecular dynamic simulations

Using classical molecular dynamic (MD) simulations the thermal conductance and the corresponding temperature profile over the junction can be calculated by means of reverse-nonequilibrium MD (RNEMD) using periodic boundary conditions as introduced by Müller-Plathe^25^,^26^. Here we use the corresponding implementation in the classical MD package LAMMPS (Large-scale Atomic/Molecular Massively Parallel Simulator)^27^,^28^ using ReaxFF potentials^29^ where we restrict the discussion to aluminum contacts as suitable parameters for lead are not available at present^30^.

In linear response the steady-state thermal conduction *κ* for a molecular junction under a temperature gradient Δ*T* is then obtained by Fourier’s law (Eq. 2) where the temperature difference Δ*T* = *T*_*R*_ − *T*_*L*_ of the right and left electrode can be obtained from the average temperature profile across the junction in steady state and d*Q*/d*t* is determined from the cumulative kinetic energy transfer 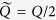 over the simulation time of the RNEMD run, here due to the periodic setup in transport direction the total energy transfer 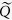 has to be divided by a factor of 2^26^. Although that in the linear response regime Δ*T* should be sufficiently small it has been shown that Eq. 2 is rather robust also with respect to larger temperature differences^11^,^31^.

**Figure 5:**
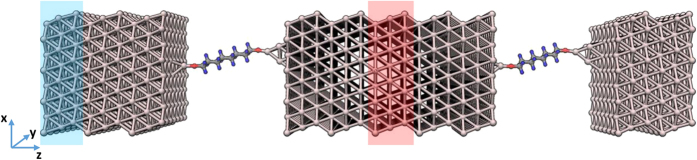
Setup used in the Müller-Plathe method to calculate the thermal conductance. The velocities of the hottest atom in the blue shaded region and the coldest in the red shaded region are exchanged.

The initial orientation of the molecule is taken from the first principles calculations and the electrodes are composed of 9 Al (111) layers in transport direction (z) with a total super cell length of 105.33 Å. Perpendicular to the transport direction the super cell is composed by 7 × 4 orthorhombic Al (111) unit cells containing 6 Al atoms with lattice parameters given by *a* = 2.86 Å, *a* = 4.96 Å, and *a* = 7.01 Å. For all calculations a time step of 1 fs is used. The initial velocities are given by a Maxwell–Boltzmann distribution at the desired average junction temperature. The system is first equilibrated for 100 ps to reach an isothermal steady state, where the system temperature is maintained using a Nose-Hoover thermostat. Then we impose a heat flux following the Müller-Plathe method by exchanging every *N* = 500 time steps the velocities of the hottest atom in the blue shaded region with the coldest atom in the red shaded region indicated in Fig. 5. In the RNEMD step the system is allowed to stabilized for 1 ns to reach a steady state, followed by a final run of 1 ns for data collection under RNEMD conditions.

To check the possible influence of finite size effects we calculated the thermal conductance for a system with reduced super cell size, namely 6 Al (111) layers in transport direction and a 5 × 3 super cell size perpendicular to the transport direction. The thermal conductance 44.5 pW/K at an average junction temperature of *T* = 307 K was found to be close to the value obtained from the larger system, suggesting that the calculations are converged with respect to the super cell size. Furthermore to confirm that we are indeed within the linear response regime it is instructive to check the dependence of the thermal conductance on the temperature difference in the electrodes. Within the RNEMD approach this can be, however, just indirectly controlled by the imposed heat flux determined by the time steps *N* between velocity exchanges. Therefore we increase the exchange frequency to *N* = 1000 and *N* = 2000 time steps to reduce the heat flux and respectively the temperature difference in the electrodes^25^,^26^. To ensure that the system reached a steady state with a converged temperature profile we increased the simulation time *t*_s_ of the RNEMD step accordingly by a factor of 2 (*t*_s_ = 2 ns) and 4 (*t*_s_ = 4 ns) respectively. The results summarized in Table 2 show that the thermal conductance is largely independent on the temperature difference between the electrodes, thus the linear relation between the heat flux and the electrode temperature difference given by Fourier’s law holds.

## Additional Information

**How to cite this article**: Buerkle, M. and Asai, Y. Thermal conductance of Teflon and Polyethylene: Insight from an atomistic, single-molecule level. *Sci. Rep.* **7**, 41898; doi: 10.1038/srep41898 (2017).

**Publisher's note:** Springer Nature remains neutral with regard to jurisdictional claims in published maps and institutional affiliations.

## Figures and Tables

**Table 1:** Thermal conductance values of the individual spatial region across the junction as well as the total junction thermal conductance *κ*.

*κ*_*L*_	*κ*_*LM*_	*κ*_*M*_	*κ*_*RM*_	*κ*_*R*_	*κ*_tot_
27480	69	722	174	27480	45.6

The thermal conductance is given in units of pW/K.

**Table 2:** Thermal conductance *κ* at an average junction temperature *T* calculated with different exchange frequencies *N*, i.e., different steady state temperature gradients Δ*T*.

*N*	*t*_s_ [ns]	Δ*T* [K]	*T* [K]	*κ* [pW/K]
500	2	213	311	45.1
1000	4	127	310	44.0
2000	8	78	301	41.3

Where the simulation time *t*_s_ of the RNEMD step was increased accordingly.
